# Generating user-driven patient personas to support preventive health care activities of rural-living unattached patients

**DOI:** 10.1016/j.pecinn.2024.100274

**Published:** 2024-03-13

**Authors:** Lindsay Burton, Kathy L. Rush, Cherisse L. Seaton, Eric P.H. Li, Kendra Corman, Charlene E. Ronquillo, Selena Davis, Mindy A. Smith

**Affiliations:** a School of Nursing, University of British Columbia, Okanagan, Kelowna, BC V1V 1V7, Canada; b Faculty of Management and Principal's Research Chair (Tier 2) in Social Innovation for Health Equity and Food Security, University of British Columbia, Okanagan, Kelowna, BC V1V 1V7, Canada; c Department of Family Practice, University of British Columbia, Vancouver, British Columbia, Canada; d Patient Voices Network, British Columbia, Canada and Department of Family Medicine, Michigan State University, East Lansing, MI, United States

**Keywords:** Preventive medicine, Primary health care, Persona development, Personas, Cluster analysis

## Abstract

**Objective:**

This study created personas using quantitative segmentation and knowledge user enhancement to inform intervention and service design for rural patients to encourage preventive care uptake.

**Methods:**

This study comprised a cross-sectional survey of rural unattached patients and a co-design workshop for persona development. Cross-sectional survey data were analyzed for meaningful subgroups based on quartiles of preventive care completion. These quartiles informed “relevant user segments” grouped according to demographics (age, sex), length of unattachment, percentage of up-to-date preventive activities, health care visit frequency, preventive priorities, communication confidence with providers, and chronic health conditions, which were then used in the workshop to build the final personas.

**Results:**

207 responses informed persona user segments, and five health care providers and 13 patients attended the workshop. The resulting four personas, included John (not up-to-date on preventive care activities), Terrance (few up-to-date preventive care activities), George (moderately up-to-date preventive care activities), and Anne (mostly up-to-date preventive care activities).

**Conclusion:**

Quantitative persona development with integrated knowledge user co-design/enhancement elevated and enriched final personas that achieved robust profiles for intervention design.

**Innovation:**

This project's use of a progressive methodology to build robust personas coupled with participant feedback on the co-design process offers a replicable approach for health researchers.

## Highlights

• Preventive care is crucial in the reduction of morbidity and mortality.
• Unattached and rural patients experience barriers to preventive care that have not been widely explored.
• Personas can be used to engage rural unattached patients in identifying needs which will inform intervention development.

## 1 Introduction

Preventive health care services aim to prevent disease (e.g., immunization) or provide early detection (e.g., cancer screening) and are associated with reduced morbidity and mortality [1], yet their use remains below recommended targets [2]. Rural patients consistently report lower preventive care utilization compared to urban counterparts [3], a disparity compounded by growing primary provider unattachment, or lacking a usual source of primary care, owing to provider shortages [4]. Few studies have targeted the unattached population, and even fewer have examined their preventive activities. [5] found a low (39.6%) prevention completion rate for rural unattached residents, with none up to date on all recommended services and only 6.7% up to date on 75% of the recommendations. Males tended to have fewer completed preventive activities and fewer priorities than females. Further, rural unattached patients remained unattached for long periods of time, ranging from one (16%) to five or more years (20%); importantly, the longer the unattachment, the lower the proportion of completed prevention activities [5].

Designing interventions that promote the uptake of preventive services for rural unattached patient service users requires positioning them at the center of the design process. There are a number of approaches to user-centred design that have evolved over the past two decades, but among those that have received growing attention in the field of health services research is the use of personas [6]. Personas are fictitious representations of groups of real users who behave similarly [7,8]. The use of personas recognizes that there is no average user but rather a mixture of certain types of users with unique characteristics [9]. Personas offer a powerful tool for distilling user data into easily communicable representations, fostering shared understanding among stakeholders across various organizational levels, and facilitating decision-making processes by providing memorable, humanized insights into user needs and preferences [10]. By better understanding their needs, preferences, feelings, and motivations, personas have proven to be valuable in tailoring and personalizing health service interventions to specific target populations, such as those with irritable bowel syndrome, HPV, and coronary heart disease [[11], [12], [13]] and enhancing service delivery [10]. While personas are typically applied to technological development, they have also become a tool for broader applications within the health intervention design and decision-making process [10,14]. Persona development begins with identifying significant patterns in users and adapting those into a meaningful archetype representing a broad section of users through qualitative, quantitative, or mixed methods [7,15,16]. Patterns emerge from combinations of identified relevant variables, such as activities, attitudes, aptitudes, motivations, and skills [7]. In persona methodology, patterns can be identified through segmentation analysis to designate “relevant user segments,” which are then enhanced with more in-depth detail, such as pictures and quotes [17,18].

Quantitative strategies for persona development using existing data offer accessible approaches for employment by health care service teams [18]. However, few quantitative developed personas have included targeted end users in the final step of enhancing user segments, which could result in disconnection between the personas and end users [18]. Further, often vague reporting of the persona development process in the literature has limited the rigour and replicability of development processes. Therefore, this study aimed to 1) create a set of personas for use in designing interventions for unattached rural patients to support and encourage preventive care uptake, 2) detail the persona development process for use in similar studies, and 3) reflect on learnings from the application of persona methodology to a health services context.

## 2 Methods

### 2.1 Design

This study used a three-step, sequential design process for iterative persona development to inform the development of an intervention to promote preventive care uptake in rural unattached patients. Persona development comprised segmentation using a quantitative rural unattached patient survey followed by a persona co-design workshop with the research team and knowledge users. Following the co-design workshop, knowledge users and research team members were invited to share feedback and reflections on the persona development process. Rurality was defined as living somewhere with a population of less than 20,000 [19].

### 2.2 Step one: quantitative segmentation

This step comprised a cross-sectional survey that gathered data on the preventive activity behaviours, priorities, and influences on preventive care utilization of unattached rural patients.

#### 2.2.1 Recruitment

Rural patients were recruited using social media advertisements, invitations on community Facebook pages, and invitations to unattached patients receiving care in clinics serving unattached patients (distributed through a patient newsletter). Recruitment venues were distributed provincially to ensure representation from each health authority region. Invitations included a link to the online survey and consent form. Recruitment took place between July 2022 and September 2022. Patients completing the survey were asked to indicate their willingness to be contacted to participate in the co-design workshop. Participants completing the survey were entered into a draw for a gift certificate valued at 50 CAD.

#### 2.2.2 Data collection

Patients were invited to complete an online survey using UBC-hosted Qualtrics with questions related to seven key content areas: a) attachment factors (e.g., the reason for unattachment, length of unattachment) [5], b) clinic usage factors (e.g., length of clinic use, number of clinic visits, types of care received at the clinic – small, routine problems or urgent care, prevention/screening care), c) health history, d) satisfaction with care [20], e) preventive activity completion (i.e., self-reported preventive activity compared to age and sex recommendations), f) preventive services use self-efficacy scale (PRESS) [21], g) perceived gaps in care (e.g., follow-up, provider continuity, referrals, preventive care), and h) preventive service initiation preferences (e.g., provider, patient, both, public health, other agencies).

#### 2.2.3 Analysis

Patient data were analyzed descriptively using SPSS 28 (IBM Corp, 2021). Survey respondents were first grouped by quartile to identify groups of unattached patients based on how up-to-date they were on preventive health care activities. This was determined based on the proportion of relevant prevention activities they had completed. Subsequently, descriptive analyses were completed and used to determine relevant user segments in characterizing and distinguishing the quartile groups according to age, prevention service priorities, PRESS scores [21], and length of unattachment. These user segments were presented in tables for further expansion during the co-design workshop and will be described later (see Table 1).

**Table 1:** User Segments of Persona characteristics.

Characteristics	Persona User Segments			
	Persona 1	Persona 2	Persona 3	Persona 4
Age range	40–50	30–40	60–70	55–65
Sex	Male	Female	Male	Female
Unattached time	5+ years	About 3 years	About 1 year	Under 1 year
Presence of chronic health conditions	None currently	None currently	Has one or more	None currently (concerned about)
Prevention activity completion	Less than 25% (not up to date)	26–50% (mostly not up to date)	51–75% (moderately up to date)	76–100% (mainly up to date)
Doctor communication self-efficacy	Low (less confident)	Low to moderate	Moderate	Moderate to high (more confident)
Prevention activity self-efficacy^1^	Low (less confident)	Low to moderate	Moderate	Moderate to high (more confident)
Prevention activity priorities	Very few	Moderate amount	Moderate amount	Moderate amount
Health care visits	Last visit over 1 year ago	1–3 time per year	3–4 times per year	1–4 times per year

*Note:* Characteristics are derived from general trends in quantitative survey data from participants separated by prevention activity completion percent.

1 Includes self-efficacy related to getting vaccines and getting preventative screenings/tests done.

### 2.3 Step two: co-design workshop

Step Two used a half-day co-design workshop bringing together knowledge users and the research team to enrich the user segments into the final personas [17]. Knowledge users included rural unattached patients and health care providers experienced and interested in prevention and or unattached rural patients.

#### 2.3.1 Recruitment

Prospective unattached patients who indicated their willingness (Step One survey) to be contacted for a persona co-design workshop received an email invitation from the team's research coordinator to participate in Step Two. Health care providers were recruited using convenience sampling and snowball strategies leveraging the research team's network connections. Interested knowledge users were sent an electronic consent to sign, confirmation of the persona workshop time, instructions for joining the online meeting, and the Zoom link. Patient participants were remunerated for their time at 25 CAD /hour and industry standard rates were applied for health care providers; one health care provider declined remuneration.

#### 2.3.2 Procedure

**Fig. 1:**
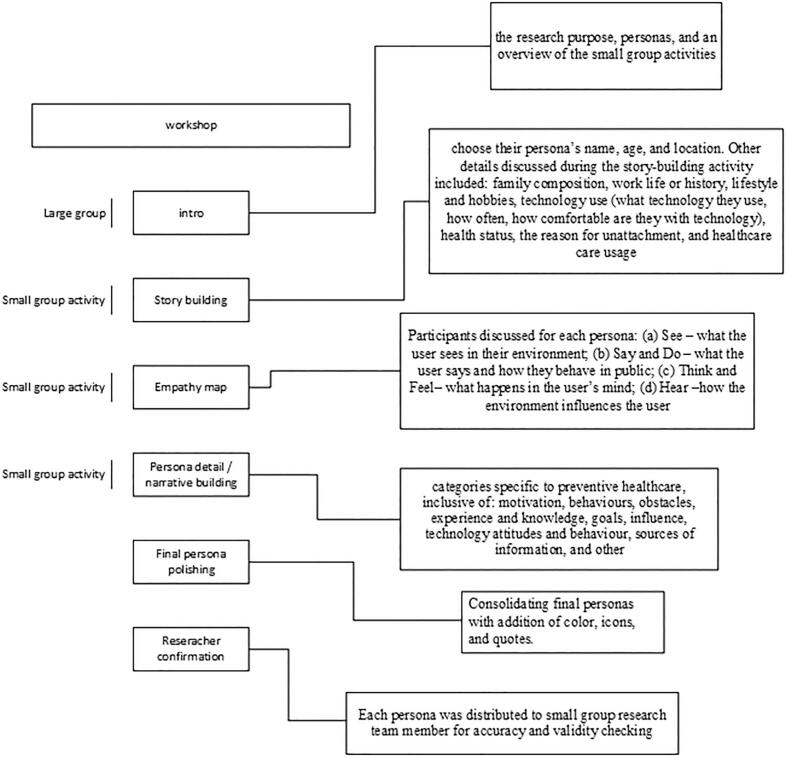
Workshop procedure.

Unattached patients and providers participated in a half-day virtual workshop co-led by research team members with expertise in persona development and prevention (Fig. 1). A systematic approach was adopted to prepare for the co-design workshop. The three activities were purposefully sequenced to build on each user segment's preventive practices and basic demographic information to provide a well-rounded profile of each user sub-group to inform design intervention development. It began with general personal information about users' backgrounds as the basis for participants assuming the mind of their evolving persona, which in turn facilitated focused attention on aspects of preventive practices to inform intervention design. To refine the workshop's delivery, a practice session involving all research team members was conducted prior to the actual workshop, ensuring a seamless and effective execution of the planned activities.

All participants received a pre-workshop package with user segments (presented in Table 1), background preparatory questions, and study information. The workshop included small- and larger-group activities. Small groups, which were facilitated by two team members (one lead and one support/note taker), generated details, descriptions, and behaviours for each persona. At the end of each small group activity, the large group convened to summarize progress on each group's persona and allow between-group feedback, which was facilitated by one team member. Small group activities were performed using online whiteboards (Zoom), allowing all participants to visualize persona components and aid in promoting discussion among the group.

The workshop began by giving participants context on the research purpose, personas, and an overview of the small group activities before breaking into small groups, that were each assigned one user segment that had been generated from Step One. The three small-group sequential activities to enrich the user segments were story-building, empathy mapping, and final persona description (Appendix A). The objective of starting with story-building was to generate comprehensive background information for each persona, enabling a deeper understanding of their history and context. Unlike traditional storytelling or scenario-based personas, which often focus on hypothetical future scenarios or specific situations [22,23], the story-building activity prioritized the development of historical narratives for each persona. This approach provided researchers and participants with an understanding of the persona, thus laying a strong foundation for subsequent persona development activities.

The second activity, empathy maps, served to get participants into the mind of their persona. The empathy map activity guided participants through four quadrants, “Think and Feel,” “See,” “Say and Do,” and “Hear” [24], to build the internal life of each persona that reflected and expanded upon the quantitative user segments. This activity encouraged participants to empathize with personas, where differing experiences or behaviours can still be understood from the perspective of the persona. Further, empathy mapping as a component of persona development has shown benefits such as flexibility, efficiency, and usefulness in guiding inexperienced participants through the persona creation process [25].

Following the empathy mapping, the final activity was persona description where participants were asked to deepen their personas using categories specific to preventive health care. Categories for the persona descriptions were chosen to reflect background and personality of the persona as well as health services design considerations (e.g., preventive care goals, motivation), common in other persona descriptions [16]. Throughout the process, research team facilitators actively tried to draw on all participants perspectives and participants were encouraged to make suggestions. When multiple options were discussed, group consensus was sought with facilitators actively constructing the personas using whiteboards during the workshop. Patients enriched the personas with personal experiences, local contexts, barriers, and frustrations. Health care providers added expert knowledge and experiences based on knowledge of rural unattached patients and preventive care needs and gaps. Following the workshop, final personas were consolidated by a team member (KC) who reviewed all recordings to ensure accuracy of the recordings to what the groups had included on the whiteboards and polished the whiteboard personas, adding color, icons, pictures, and quotes. Because the story building and persona development activities were structured, with participants enriching each persona in real-time, there was minimal need for synthesis post-workshop. The quotes included with each persona were largely drawn as compilations from the empathy mapping exercise, and adjusted to first person, when needed. Each final persona was then distributed to research team members for member checking and validation. The workshop was audio and video recorded, videoconference chats were saved, and all whiteboards were downloaded for a data audit trail.

### 2.4 Persona development process feedback and reflections

All knowledge users were emailed an investigator generated, online, 17-item survey two weeks after the workshop inviting feedback related to satisfaction, preparation and design, experience, and future workshops. The survey used a combination of Likert scales, multiple selection, and open-ended questions (See Appendix B). Researcher team reflections were gathered during the workshop debrief and in follow-up email correspondence.

## 3 Results

### 3.1 Step one: user segmentation

Initial survey respondents (*n* = 207) ranged in age from 19 to 80 years, with a mean age of 47 (SD = 14.3) years, and were mostly female (74%). Participants were, on average, up to date on 51% of preventive activities. Seventeen percent of participants had been unattached for 6 months or less, 53% 6 months to 5 years, and 30% had been unattached for over five years. Participants reported being unattached because they had no desire to be attached (4%), their doctor retired or left their community (39%), and no doctors were accepting new patients (60%). When participants required non-urgent or routine health care, they received care at walk-in clinics (43%), urgent care clinics (8%), the emergency department (21%), or did not seek care (29%).

User segmentation was calculated from 207 responses to the online survey. The relevant user segments were derived based on characteristic trends from participants who were 0–25% (*n* = 38), 26–50% (*n* = 66), 51–75% (n = 66), and 76–100% (*n* = 37) up to date on preventative health care activities. The user segments that contained the guiding information and attributes for each persona are presented in Table 1. Table 1 presents the quartile clusters grouped according to demographics (age, sex), length of unattachment, percentage of up-to-date preventive activities, health care visit frequency, preventive priorities, communication confidence with providers, and chronic health conditions. It is important to note that because each of the four groupings of participants contained relatively similar percentages of male participants (range: 21.6% to 28.8%), and male participants were underrepresented in the sample overall, it was collectively decided to alternate the sex of each user segment.

### 3.2 Step two: user-enriched personas

The persona workshop session took place in Fall of 2022. The workshop was attended by five health care providers (clinical nurse specialist, nurse practitioner, and three primary care physicians) and 13 rural unattached patients. Female patients were mainly represented (85%). Two males who had previously signed up for the workshop did not attend without reason. Patient's ages were 61–70 years (*n* = 5), 51–60 years (*n* = 2), 41–50 years (*n* = 4), and 31–40 years (n = 2). The majority of patients were Caucasian (*n* = 11).

The co-design process for persona development was organized into 4 smaller sub-groups for breakout discussions and each sub-group assigned one of the persona user segments to work with throughout the workshop. Small group composition was assigned after participants signed up but before the session began to ensure age, sex, and representation from each health authority in each group. Each small group also had at least one health care provider. Although the plan had been to assign one male to each small group, with two not attending the workshop, only two of the persona small groups (both the male personas) included male patients. Small groups had four to five participants, along with two to three research team members. In one small group, the health care provider had to leave before the third small group activity. Participants and researchers worked together during the workshop to complete activities and reach a consensus on the final details of each persona.

**Fig. 2:**
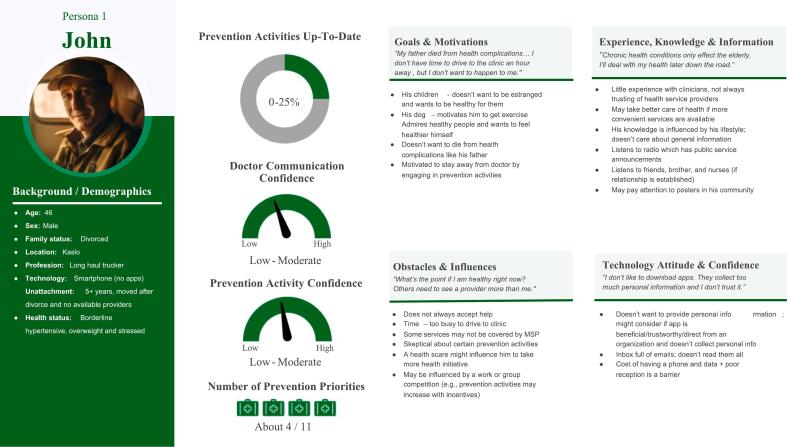
Persona John.

**Fig. 3:**
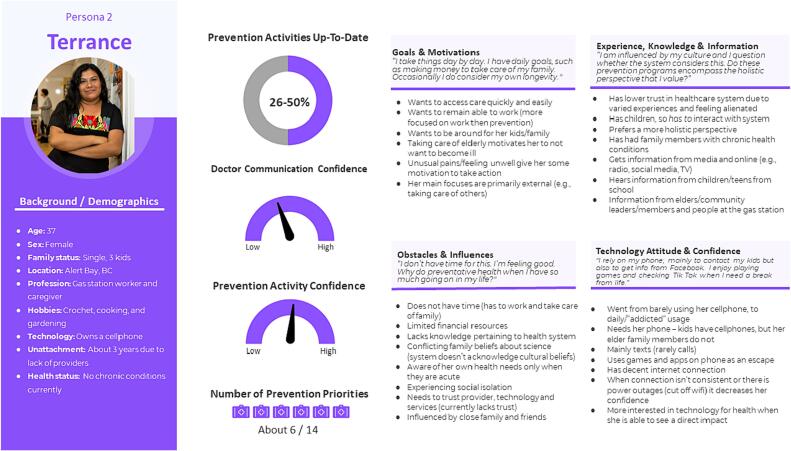
Persona Terrance.

**Fig. 4:**
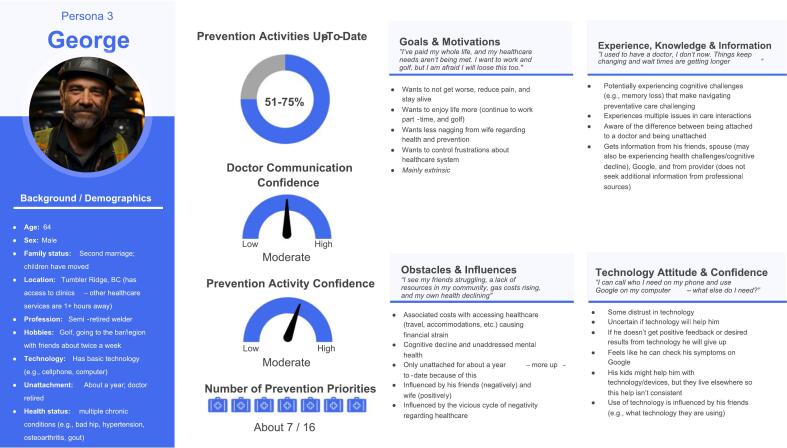
Persona George.

**Fig. 5:**
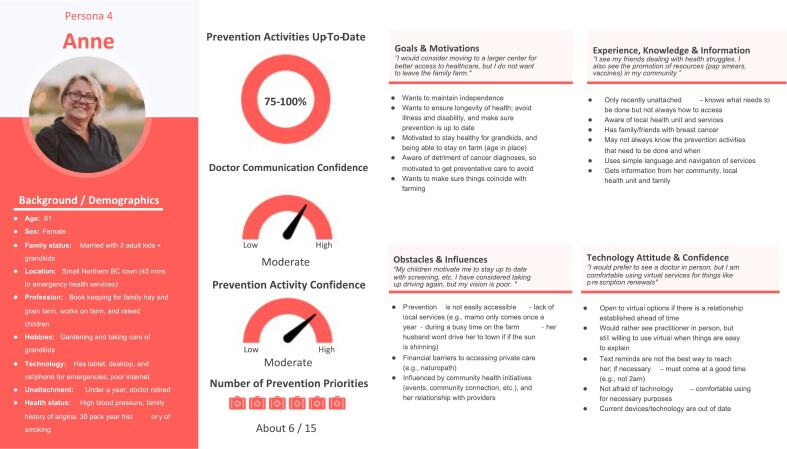
Persona Anne.

The final four enriched personas that resulted included: John (not up-to-date on preventive care activities) (Fig. 2), Terrance (few up-to-date preventive care activities) (Fig. 3), George (moderately up-to-date preventive care activities) (Fig. 4), and Anne (mostly up-to-date preventive care activities) (Fig. 5) that represented typical experiences and realities of rural unattached patients.

### 3.3 Reflections and participant feedback

#### 3.3.1 Knowledge users

Six knowledge users provided feedback on the persona process. Feedback and reflections were combined due to the small group size and possibility of identifying participants. Participants were either satisfied or neutral in their satisfaction with the workshop. All respondents understood workshop expectations and the meaning of personas. They found activities interesting and helpful in empathizing with personas and the combination of small and large-group activities useful in persona design. With one exception all agreed that the pace and length of the workshop were appropriate. Half found online whiteboards helpful. Four felt their participation was beneficial to developing the personas, while two disagreed. Four participants agreed they were at ease sharing their thoughts while two disagreed. Three participants reported no distress, while two expressed personal distress or discomfort when discussing the personas; one participant followed up after the session requesting removal from future studies because of the distress.

Knowledge user suggestions for future persona workshops included slowing down the workshop pace, allowing more overall workshop and debriefing time after each small-group activity, and adjusting the number of participants (both more and less). Respondents also suggested more clarification on workshop objectives and more guidance from small-group facilitators. Multiple respondents requested time during the workshop to share personal experiences of being unattached. This feedback was echoed by the research team. A follow-up knowledge user suggestion was to integrate decolonized techniques into the persona workshop and to be gender inclusive to address accessibility challenges for the diversity of the unattached population.

#### 3.3.2 Research team members

Immediately following the workshop the research team debriefed and reflected on the workshop sessions. Team members had not anticipated the strong emotions and ongoing frustrations over unattachment that permeated the discussions and activities with participants. This made it challenging in the short timeframe of the workshop to manage the emotional frustrations while staying on task to gather participant perspectives for persona development in some groups. Additionally, the mix of providers and patients in the small groups made facilitation challenging in ensuring inclusive perspectives, as providers tended to be more comfortable in expressing their perspectives, while patients were quieter and often shared only when invited to do so.

## 4 Discussion and conclusion

### 4.1 Discussion

This work contributes to the growing body of knowledge related to persona use in health services intervention research, with a recent review reporting 20% (21/105) of papers on the use of case persona design applied to the health care domain [26]. It is the first usage of personas to categorize unattached patients and their preventive care using a sequential process of quantitative user segment creation followed by knowledge user co-design.

Addressing the study's first aim, this novel approach that started with quantitative survey data to provide a beginning objective look at each persona's preventive care activities and practices was incomplete until the workshop brought the four rural unattached personas to life through sequential activities. This design process produced four distinct and well-rounded personas, reflecting the rural unattached patient population as a mixture of sub-groups with specific patterns of needs, motivations, and preventive uptake.

This study's second aim, was to provide a detailed description of our process. This process, through empathy maps and story building, which have been well established in previous persona studies [26], was designed to encourage participants to maintain some objectivity while infusing the persona with their own experiences of preventive care and unattachment. The intent of the persona process was to have participants who represented the rural unattached population more fully develop the persona to which they were assigned while maintaining distance. However, findings from this study suggest that, depending on the phenomena, balancing distance and objectivity with personal experience can be particularly challenging.

The importance of patient voices in developing personas for design of service program and interventions was highlighted in the process. A lack of end user involvement in persona development and narrative structuring is an identified limitation in previous studies [27,28]. While co-design personas have been previously used, no description of the persona development process was provided [29], limiting the replicability of their approach. This further highlights critiques of persona methodologies as lacking verifiability [17,18]. It is therefore all the more critical to provide detailed reporting of persona methods [28], as this paper has done.

Our final aim built on existing persona methodologies, that combined both a quantitative survey to define the user segments and a workshop of rural unattached patients and providers to enhance the personas, provided a robust pool of the user population for the process of persona development. While participation in providing feedback on the persona workshops was low, it provided invaluable feedback on the process that will strengthen future persona development process designs [30].

If the persona development process is to drive interventions, hearing from participants is essential to ensuring the process is not creating barriers to their engagement in the process. However, future work should emphasize robust evaluation of the persona development process to advance the quality of the process and maximize participant engagement in ensuring an inclusive tailored approach to planning and design of preventive services for rural unattached patients. Beyond the persona development process is the need for evaluating the process of persona implementation in actual service design work.

### 4.2 Strengths and limitations

This study is not without limitations. Firstly, while it is common in such workshop sessions to mix both patients and health care providers, the power differential because of providers' positions of authority, could have influenced patients' participation, and their willingness to share negative experiences or to challenge dominant provider perspectives. While researchers made efforts to ensure all individuals in the sessions were active participants, separating providers and patients for these sessions should be considered in future work. Other research similarly recommends using participants of “similar backgrounds” [31,32], however their persona development process did not use data driven segmentation prior to persona creation, rather grouped participants of similar experience together in twos to build personas. Secondly, information on technology attitudes and confidence related to preventive care to enhance the personas came only during Step Two workshops from unattached patients and provider expertise and not from the survey. The use of the larger survey participant pool may have informed the user segments differently than the workshop participant pool and yet the workshop participants had the benefit of “knowing” their persona more holistically to consider, and better reflect, the person's technology needs and practices. Finally, the decision to use online workshop is not without limitations. Participants were required to have a highspeed stable internet connection to fully participate which may have excluded some rural individuals. However, the use of online workshops facilitated the participation of individuals from multiple locations as well as provided a suite of tools for facilitating the persona co-design (e.g., Zoom whiteboards).

### 4.3 Innovation

Personas are an established user-centred design methodology; however, their use in health services intervention design is still relatively new [26]. Our study builds on existing strategies while providing a vivid process description to inform future research. While other persona development strategies have combined both quantitative and qualitative elements [33], few quantitative personas have included targeted end users in the final step of enhancing user segments. This novel approach contributes greater insights into how potential end-users can best be engaged to optimize and enrich the quality and completeness of personas. The outcome was a fulsome profiling of four rural unattached patient personas that can inform more tailored preventive care services interventions. Indeed, after this research, the four personas were used to guide small group discussions for a project on designing preventive screening and episodic care services for rural attached patients by a virtual clinic. This additionally demonstrates their use as a powerful knowledge translation tool [32].

The addition of co-design with end users, often missing from service design, positioned unattached patients as active participants in shaping personas rather than as passive objects of information [34]. This active participation in persona creation also provided a deeper understanding of the rural unattached population and the emotional frustration experienced, that resonates with other studies and echoed well-known disparities in rural areas compounded by service scarcity [35]. However, it also illuminated deeper issues in the user-centred design process of persona creation for highly sensitive topics, that has had limited examination. Our findings are also contrary to previous work, which found personas facilitated more open discussion of sensitive topics among HIV positive gay men [36]. However, the topic for developing personas in that population was around smoking cessation, this may not have had the same emotional charge and stress as has been found in unattached patients [37]. The findings from this work point to the need for integrating mitigating elements into the persona creation process, to address emotional distress that may influence the goals of the process.

### 4.4 Conclusion

As the health sector is moving towards a more human-centric and needs-based service approach, the present study offers health researchers and practitioners an understanding of the benefit of engaging patients in persona development that represent a diversity of patient segments. The multi-dimensionality and storytelling approach of our persona study showcased the importance of contextualizing patients' experiences in a narrative style. Further, the immediate uptake of these personas for use in health care service design demonstrates the utility in translating complex and diverse patient needs in an accessible manner.

## CRediT authorship contribution statement

**Lindsay Burton:** Writing – review & editing, Writing – original draft, Methodology, Investigation, Funding acquisition, Formal analysis, Conceptualization. **Kathy L. Rush:** Writing – review & editing, Writing – original draft, Supervision, Methodology, Funding acquisition, Formal analysis, Conceptualization. **Cherisse L. Seaton:** Writing – review & editing, Writing – original draft, Methodology, Formal analysis, Conceptualization. **Eric P.H. Li:** Writing – review & editing, Investigation, Funding acquisition, Conceptualization. **Kendra Corman:** Writing – review & editing, Formal analysis, Data curation. **Charlene E. Ronquillo:** Writing – review & editing, Methodology, Data curation, Conceptualization. **Selena Davis:** Writing – review & editing, Conceptualization. **Mindy A. Smith:** Writing – review & editing, Writing – original draft, Investigation, Funding acquisition, Conceptualization.

## Declaration of competing interest

The authors declare that they have no known competing financial interests or personal relationships that could have appeared to influence the work reported in this paper.

## Appendix A Supplementary data

Persona Workshop Whiteboard Templates Image 1

Supplementary material 2 Persona Worshop Evaluation Survey Image 2
