# Supplementary material for: Generating user-driven patient personas to support preventive health care activities of rural-living unattached patients
Source: PEC Innov. 2024 Mar 13;4:100274. doi: 10.1016/j.pecinn.2024.100274 (PMC10973187; doi:10.1016/j.pecinn.2024.100274)
Supplement: Supplementary file 1 — Persona Workshop Whiteboard Templates [file mmc1.docx]

Appendix B: Persona Workshop Whiteboard Templates


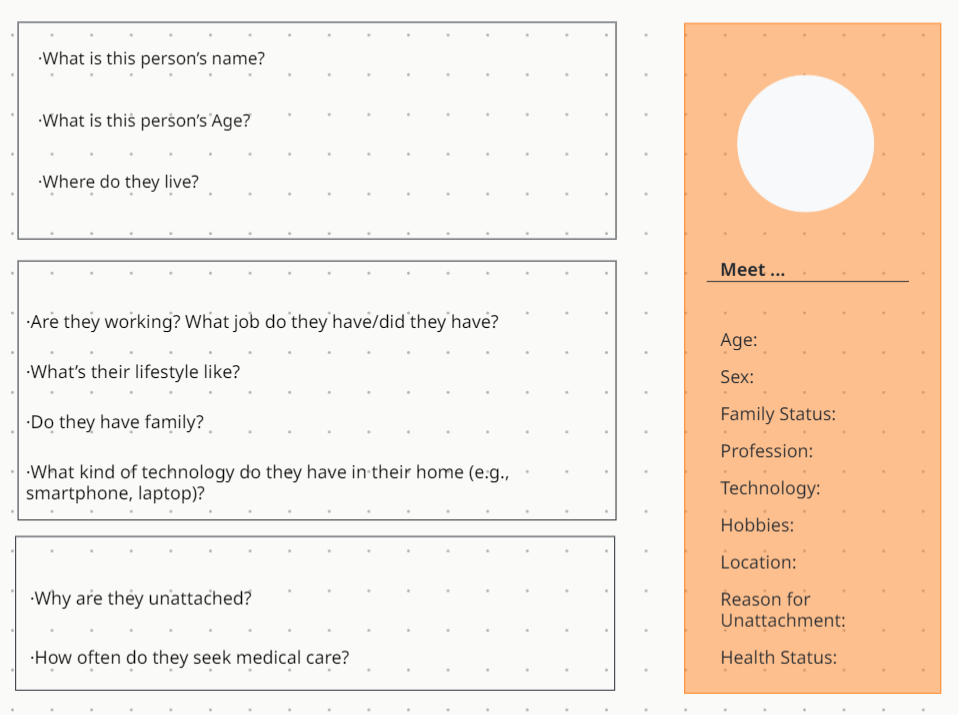


Story Building


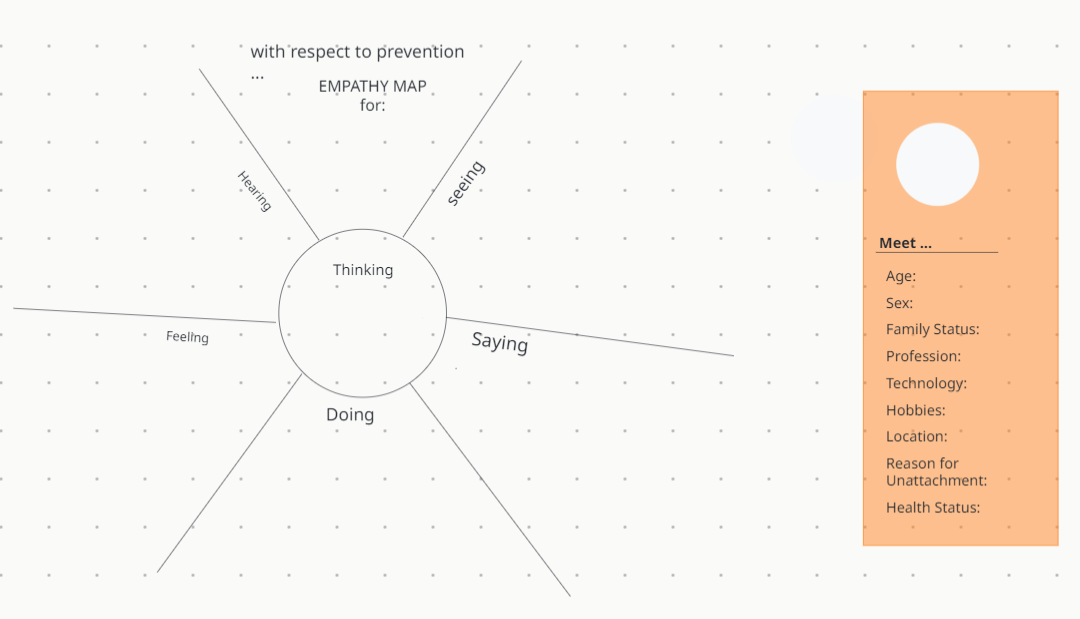


Empathy Map


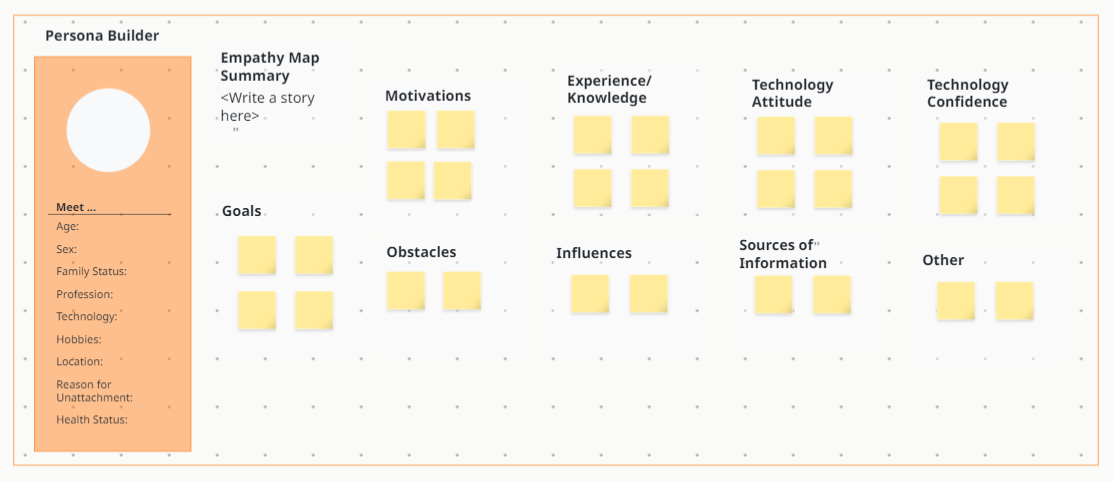


Persona Description
