# Supplementary material for: Generating user-driven patient personas to support preventive health care activities of rural-living unattached patients
Source: PEC Innov. 2024 Mar 13;4:100274. doi: 10.1016/j.pecinn.2024.100274 (PMC10973187; doi:10.1016/j.pecinn.2024.100274)
Supplement: Supplementary file 2 — Supplementary material 2 Persona Worshop Evaluation Survey [file mmc2.docx]

**Appendix B: Persona Feedback Survey**

# INSTRUCTIONS

Please select your response to the items. Rate aspects of the persona workshop on a 1 to 5 scale:

1 = "Strongly disagree," or the lowest, most negative impression 3 = "Neither agree nor disagree," or a neutral impression

5 = "Strongly agree," or the highest, most positive impression

Choose N/A if the item is not appropriate or not applicable to this workshop. Your feedback is sincerely appreciated. Thank you.

**PERSONA WORKSHOP SATISFACTION**

|  | Very Unsatisfied (1) | | |  | Very Satisfied (5) | |  |
| --- | --- | --- | --- | --- | --- | --- | --- |
| 1. How would you rate your overall satisfaction participating in the session? | | 1 | 2 | 3 | 4 | 5 | N/A |

**PERSONA WORKSHOP PREPARATION AND DESIGN**

|  | Strongly disagree (1) | | |  | Strongly agree (5) | |  |
| --- | --- | --- | --- | --- | --- | --- | --- |
| 1. I was well informed and clear on the objectives of this persona workshop. | | 1 | 2 | 3 | 4 | 5 | N/A |
| 2. I understood what would be taking place during the persona workshop. | | 1 | 2 | 3 | 4 | 5 | N/A |
| 3. I was confused about the meaning of a persona. | | 1 | 2 | 3 | 4 | 5 | N/A |

| 5. The persona workshop activities were engaging. | 1 | 2 | 3 | 4 | 5 | N/A |
| --- | --- | --- | --- | --- | --- | --- |

| 6. The activities in this persona workshop gave me sufficient background to put myself in the persona’s shoes. | | | 1 | | 2 | | 3 | | 4 | | 5 | | N/A |
| --- | --- | --- | --- | --- | --- | --- | --- | --- | --- | --- | --- | --- | --- |
| 7. Reviewing other group’s activities during the persona workshop was helpful to the process | | | 1 | | 2 | | 3 | | 4 | | 5 | | N/A |
| 8. The pace of this persona workshop was appropriate (i.e., you did not feel rushed, nor did you feel like you had *too* much time). | | | 1 | | 2 | | 3 | | 4 | | 5 | | N/A |
| 9. The total length of the workshop (3 hours) was appropriate (was not too short or too long) | | | 1 | | 2 | | 3 | | 4 | | 5 | | N/A |
| 10. The use of the whiteboards/sticky notes helped in developing the persona | | | 1 | | 2 | | 3 | | 4 | | 5 | | N/A |
| **PERSONA WORKSHOP EXPERIENCE** | | |  | |  | |  | |  | |  | |  |
| I felt like my participation in the persona workshop was valuable | 1 | 2 | | 3 | | 4 | | 5 | | N/A | |  |  |
| I felt comfortable providing input | 1 | 2 | | 3 | | 4 | | 5 | | N/A | |  |  |
| I experienced personal distress or discomfort when discussing the persona | 1 | 2 | | 3 | | 4 | | 5 | | N/A | |  |  |
|  | | |  | |  | |  | |  | |  | |  |

**FURTURE PERSONA WORKSHOPS**

1. How would you improve this workshop? (Check all that apply.)

Provide better information before the workshop.

Clarify the workshop objectives.

Reduce the content covered in the workshop.

Increase the content covered in the workshop.

Make workshop activities more stimulating.

Improve workshop organization.

Slow down the pace of the workshop.

Speed up the pace of the workshop.

Allot more time for the workshop.

Shorten the time for the workshop.

___Provide more opportunities to review the personas created by other groups.

___Allow time to debrief or share personal experiences during the workshop.

___Have fewer participants in each breakout room

___Have more participants in each breakout room

___Have instruction/guidance from facilitators

___Have less instruction/guidance from facilitators

1. What other improvements would you recommend in this workshop? ____________
2. What is least valuable about this workshop? ________________________
3. What is most valuable about this workshop? _______________________

Do you have any additional comments or suggestions about the workshop?

________________________________________________________________________________________________________________________
